# The efficacy and safety of pharmacological treatments for lymphangioleiomyomatosis

**DOI:** 10.1186/s12931-020-1316-3

**Published:** 2020-02-14

**Authors:** Qi Wang, Mengqi Luo, Bo Xiang, Siyuan Chen, Yi Ji

**Affiliations:** 1 Division of Oncology, Department of Pediatric Surgery, West China Hospital of Sichuan University, #37# Guo-Xue-Xiang, Chengdu, 610041 China; 2 State Key Laboratory of Oral Diseases, National Clinical Research Centre for Oral Diseases, West China Hospital of Stomatology, Sichuan University, Chengdu, China; 3 Pediatric Intensive Care Unit, Department of Critical Care Medicine, West China Hospital of Sichuan University, Chengdu, China

**Keywords:** Lymphangioleiomyomatosis, Pharmacological treatments, Efficacy, Safety, Meta-analysis

## Abstract

**Background:**

Lymphangioleiomyomatosis (LAM) is a rare, low-grade multisystem neoplastic disease**.** Most LAM patients are at a high risk of losing lung function at an accelerated rate and developing progressive dyspnea. Recently, several studies have reported their experience with pharmacological treatments for LAM. Therefore, we conducted a systematic review and meta-analysis to assess the efficacy and safety of these therapies.

**Methods:**

PubMed (Medline), EMBASE, Cochrane Library, Web of Science and EBSCO Host were searched (until March 31, 2019) for eligible prospective studies regarding LAM patients treated with pharmacological treatments. Random effect models were used for quantitative analysis.

**Results:**

Fourteen prospective studies regarding five pharmacological treatments (including sirolimus, everolimus, doxycycline, triptorelin, and a combination therapy of sirolimus and hydroxychloroquine) were enrolled in our systematic review, and ten of them were used for the meta-analysis. Seven prospective studies reported that sirolimus was effective at improving or stabilizing lung function and alleviating renal angiomyolipoma (AML) in LAM patients. Subsequent quantitative analyses showed that during sirolimus treatment, the pooled values of lung function and 6-min walk distance (6MWD) were not significantly changed (*P* > 0.05), with the pooled response rate of AML being 0.62 (95% confidence intervals [CIs]: 0.43 to 0.82, *I*^*2*^ = 65%). Regarding everolimus, three prospective studies reported similar effects to those of sirolimus with regard to preserving lung function and reducing AMLs. The meta-analysis showed that the changes in lung function during everolimus treatment were not statistically significant (*P* > 0.05), while the pooled response rate of AML was 0.78 (95% CI: 0.68 to 0.88, *I*^*2*^ = 8%). Neither the qualitative nor the quantitative results confirmed the benefits of doxycycline or triptorelin treatment, and the effects of the combination therapy were unclear in LAM patients. Most of the adverse events during pharmacological treatments were low or moderate grade and tolerable.

**Conclusions:**

Overall, sirolimus and everolimus were recommended for the treatment of LAM because they could stabilize lung function and alleviate renal AML. Doxycycline and triptorelin were not recommended for the treatment of LAM because no beneficial outcomes were consistently observed. The efficacy and safety of combination therapy remain to be further explored.

## Background

Lymphangioleiomyomatosis (LAM) is a rare, low-grade multisystem neoplastic disease characterized by cystic lung destruction, chylous fluid accumulation (pleural or ascitic), angiomyolipomas (AMLs) and lymphangioleiomyomas [1, 2]. LAM occurs sporadically (sLAM) or is associated with tuberous sclerosis complex (TSC). It affects adult females almost exclusively, with a prevalence of approximately five per million, although LAM has also been reported in adult males and children [3–5]. In LAM, the progressive cystic destruction of the lung can lead to recurrent pneumothorax and dyspnea, resulting in lung function declines at rates of 2–4 times or more compared with the typical age-related decline rate [6, 7]. In many patients, dyspnea with daily activities and hypoxia requiring oxygen support can also occur within 10 years of symptom onset [8]. For LAM patients in the end stage, lung transplantation is the only treatment option [6, 7]. Although AMLs are benign and usually asymptomatic, they may enlarge and bleed, which can lead to chronic kidney disease or require urgent treatment [9]. However, LAM may recur in transplanted lungs [10, 11], and patients with AML often develop new lesions and relapse after treatment [12].

Genetic studies have revealed that patients with LAM have biallelic inactivating mutations in the TSC-1 or TSC-2 gene [13]. Loss of TSC gene function activates the mammalian target of rapamycin (mTOR) signaling pathway, which is a key regulator of cell growth, motility, and survival, resulting in the proliferation of LAM cells [14]. The lungs and lymphatics of LAM patients are infiltrated with LAM cells [15]. Although LAM has a benign histological appearance, LAM cells can circulate in the blood and lymphatic fluids [16]. Moreover, LAM cells can express vascular endothelial growth factor D (VEGF-D), which may facilitate the migration of LAM cells to lymphatic vessels and promote metastatic spread [17, 18].

In addition to lung transplantation for LAM, hormonal therapies (e.g., progestins, gonadotrophin-releasing hormone analogs, and antiestrogen therapies) have previously been the main initial treatment options [19–21]. Doxycycline, a tetracycline antibiotic and matrix metalloproteinase (MMP) inhibitor, has also been reported to be effective in the improvement of lung function in LAM patients. Clinical trials have been performed to evaluate the exact effect of doxycycline [22, 23]. Interestingly, mTOR inhibitors (sirolimus and everolimus) have been reported to be effective at reducing AML volume and improving or stabilizing lung function in LAM patients [24–26]. In addition, clinical practice guideline have recommended sirolimus for LAM patients with declining lung function and problematic chylous effusions, although this recommendation is based on moderate-quality to very low-quality evidence [1]. Because LAM is an orphan disease, it is inevitable that the sample size and data collection will be limited. To date, there is still a lack of studies evaluating the efficacy and safety of all pharmacological treatments for LAM.

Therefore, we performed this literature review to present updated insights into the pharmacological treatment of LAM and to conduct a meta-analysis that assesses the efficacy and safety of these therapies.

## Methods

### Search strategy

Studies were identified by searching PubMed (Medline), EMBASE, Cochrane Library, Web of Science and EBSCO Host until March 31, 2019. OpenThesis and OpenGrey were also searched for gray literature. Medical Subject Headings (MeSH) and free text words were applied during the search, including (‘Lymphangioleiomyomatosis’ OR ‘Lymphangioleiomyomatoses’ OR ‘Lymphangiomyomatosis’ OR ‘Lymphangiomyomatoses’) AND (‘Therapeutics’ OR ‘Therapeutic’ OR ‘Therapy’ OR ‘Therapies’ OR ‘Treatment’ OR ‘Treatments’). Publications were restricted to research on humans and studies written in English or Chinese. The reference lists were also searched for additional relevant studies. This systematic review was registered in the s database with the registration number CRD42019122366.

### Inclusion criteria and exclusion criteria

Studies were included if they met the following inclusion criteria: (1) any pharmacological treatment for sLAM or TSC-LAM, with or without a control group, and (2) a clear description of outcomes, adverse events (AEs) and side effects. The exclusion criteria were set as follows: (1) duplicate publications; (2) research presenting interim or extended findings regarding the same group of patients; (3) studies with a sample size of fewer than five patients; (4) publications with no detailed original data (such as abstracts, posters, conference reports, letters, case reports, reviews and meta-analyses); and (5) studies with a retrospective design.

### Data extraction

Two reviewers (WQ and LMQ) independently assessed the eligibility and risk of bias of the studies and extracted the data. Disagreements were resolved by discussion with the third investigator (JY). For each suitable study, the following data were collected using a standard Excel form: (1) general information: name of first author, publication year, study location, study design, number and diagnosis of participants, study duration, treatment name and regimen, and study outcomes; (2) baseline and follow-up efficacy data: forced expiratory volume in 1 s (FEV_1_), forced vital capacity (FVC), diffusing capacity for carbon monoxide (DLco), 6-min walk distance (6MWD), quality of life (QOL) scores, serum level of VEGF-D and response rate of AML; and (3) AEs (mentioned in at least 2 studies): types of AEs and numbers of patients with AEs. First author and corresponding author were contacted to obtain missing data when possible.

### Quality assessment

The risk of bias of the randomized controlled trials (RCTs) was assessed by the Cochrane criteria with regard to the following items: random sequence generation, allocation concealment, blinding of participants, personnel and outcome assessments, incomplete outcome data, selective reporting and other bias. The quality of single-arm trials was assessed according to the methodological index for nonrandomized studies (MINORS) [27], and the items included a clear stated aim, inclusion of consecutive patients, prospective collection of data, endpoints appropriate to the aim of the study, unbiased assessment of the study endpoint, follow-up period appropriate to the aim of the study, loss to follow-up less than 5% and prospective calculation of the sample size.

### Data synthesis and statistical analysis

In this study, the protocol was performed based on the Preferred Reporting Items for Systematic Reviews and Meta-Analyses (PRISMA) guidelines [28]. Review Manager (*version 5.3*) and *R* software were used to perform the meta-analysis. For continuous data including the 6MWD, serum level of VEGF-D, and absolute values of FEV_1_, FVC and DLco at baseline and the endpoint, we extracted the means and standard deviations (SDs) from the included articles. The means and SDs were estimated by the methods described in the Cochrane handbook [29] or were estimated from the sample size, median, range and/or interquartile range if they were not directly reported [30–32]. Moreover, we converted some data to ensure that they had the same unit of measure. The effect sizes were analyzed using a random effect model and are reported as the weighted mean difference (WMD). For dichotomous data, the pooled proportions were analyzed by *R* software with a meta-package in a random effect model. In addition, 95% confidence intervals (CIs) were reported for each measure. Heterogeneity among the included studies was evaluated by *I*^*2*^ statistics (low heterogeneity: *I*^*2*^ ≤ 25%; moderate: 25–50%; high> 75%). A sensitivity analysis omitting each included study in turn was also performed to evaluate the effects of individual studies on the statistical results. Probability values < 0.05 were considered to indicate statistical significance.

As the number of included articles about each pharmacological treatment was fewer than 10, publication bias could not be explored in this study.

## Results

### Search results and characteristics of the included studies

**Fig. 1:**
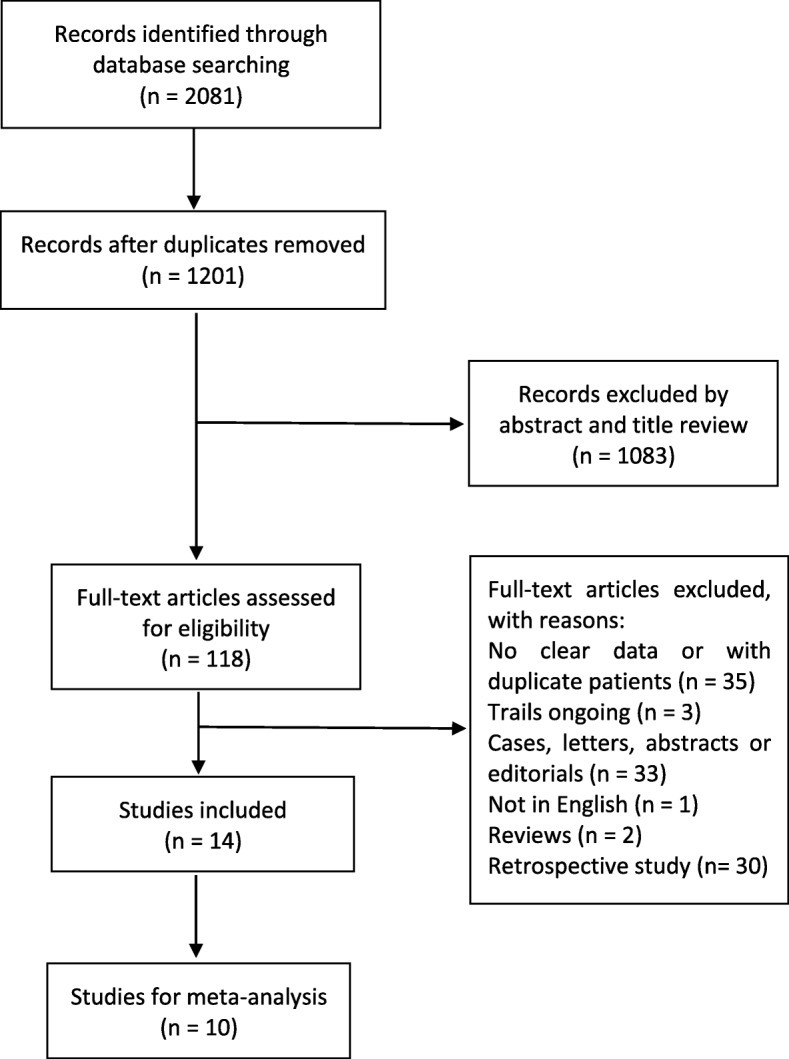
Flow chart of the study selection process. The search and screening process of eligible studies, and the number of studies at each stage.

The search and screening process for the studies is presented in Fig. 1. A total of 2081 records were identified through the initial database search. After removing duplicates and reviewing titles and abstracts, the eligibility of the remaining 118 articles was carefully assessed by a full-text review. Finally, only 14 studies met the inclusion criteria for the systematic review, including 3 RCTs [23, 25, 33] and 11 single-arm trials [9, 20, 22, 24, 34–40], of which ten studies were included in the meta-analysis [22–25, 33–36, 39, 40]. Bissler et al. reported accurate data on lung function in LAM patients treated with everolimus in an article published in 2017 [41]; these data were not available in the initial article published in 2013 [25]. Therefore, we cited these two articles as the same RCT. Because there was only one article each describing triptorelin monotherapy [20] and a combination therapy of sirolimus plus hydroxychloroquine [38], these two articles were only used for qualitative analyses. Moreover, the endpoints and/or baseline lung function values were not available from the studies by Takada et al. [37] and Bee et al. [9]. Therefore, these two articles were not included in the meta-analysis.

The characteristics of the included studies are summarized in Table 1. The included prospective clinical trials and RCTs reported five pharmacological treatments for 330 LAM patients. Among them, 46 patients in 1 RCT treatment group [33] and 164 patients in 6 single-arm trials [9, 24, 34, 35, 37, 40] were treated with sirolimus. Twenty-three patients in 1 RCT treatment group [25] and 30 patients in 2 single-arm trials [36, 39] were treated with everolimus. Twelve patients in 1 RCT treatment group [23] and 31 patients in a single-arm trial [22] were treated with doxycycline. In addition, 11 patients were reported to be treated with triptorelin [20], and 13 patients were reported to be treated with a combination of sirolimus and hydroxychloroquine [38] in single-arm trials. The quality assessments of the RCTs and single-arm trials are presented in Additional file Table A1 and Table A2.

**Table 1:** Characteristics of the included studies

Author, Publication Year, Country	Journal	Study design	Diagnosis, No. of patients	Study duration	Intervention and sample size	Treatment regimen	Study outcomes
Bissler [24] (2008) USA	N Engl J Med	Single-center, nonrandomized open-label phase 1–2 trial	LAM (*n* = 6), TSC-LAM (*n* = 12), TSC (*n* = 7),	1 year of treatment, 1 year of observation	Sirolimus (*n* = 25)	0.25 mg/m^2^ body-surface area	Renal AMLs, lung function, lung cyst volume, 6MWD, AEs, neurologic assessment.
Harari [20] (2008) Italy	Chest	Single-center, prospective phase 1 trial	LAM (*n* = 11)	3 years of treatment	Triptorelin (*n* = 11)	11.25 mg IM	Hormonal assays, pulmonary function tests, 6MWD, bone mineral density.
Dabora [34] (2011) USA	PLoS ONE	Multicenter open-label, phase 2 trial, single-arm	TSC-LAM (*n* = 21), TSC (*n* = 15)	1 year of treatment	Sirolimus (*n* = 36)	2 mg/day	AMLs, kidney cysts, skin lesions, pulmonary function, VEGF-D.
McCormack [33] (2011) USA	N Engl J Med	Multicenter, randomized, placebo-controlled study	LAM (*n* = 81), TSC-LAM (*n* = 8)	1 year of treatment, 1 year of observation	Sirolimus group (*n* = 46); Placebo group (*n* = 43)	2 mg/day	Lung function, 6MWD, VEGF-D, QOL scores, AEs.
Davies [35] (2011) UK	Clin Cancer Res	Multicenter nonrandomized open label phase 2 trial	LAM (*n* = 6), TSC-LAM (*n* = 3), TSC (*n* = 7),	2 years of treatment	Sirolimus (*n* = 16)	0.5 mg/m^2^ body-surface area	Renal AMLs, lung function, AEs and neurocognitive function.
Bissler [25] (2013) USA	Lancet	Multicenter, randomized, double-blind, placebo-controlled phase 3 trial	LAM (*n* = 5), TSC-LAM (*n* = 24), TSC (*n* = 89)	Median 38 weeks for everolimus; median 34 weeks for placebo.	Everolimus group (*n* = 79); Placebo group (*n* = 39)	10 mg/day	AMLs, skin lesion, pharmacokinetics of everolimus, pulmonary function, AEs, neuropsychological assessments, VEGF-D.
Piment a[22] (2013) Brazil	J Bras Pneumol	Single-center open-label, single-arm, interventional clinical trial	LAM (*n* = 31)	1 year of treatment	Doxycycline (*n* = 31)	100 mg/day	Pulmonary function, 6MWD, QOL, MMP-2, MMP-9, VEGF-D
Chang [23] (2014) UK	Eur Respir J	Single-center randomized placebo-controlled trial	LAM (*n* = 23)	2 years of treatment	Doxycycline group (*n* = 12); Placebo group (*n* = 11)	100 mg/day for 3 months followed by 200 mg/day for 21 months	Lung function, exercise capacity, QOL, shuttle walk distance, MMP levels, VEGF-D.
Goldberg [36] (2015) USA	Eur Respir J	Multicenter, open-label, nonrandomized, phase 2 trial	LAM (*n* = 19), TSC-LAM (*n* = 5),	Treatment for 26 weeks	Everolimus (*n* = 24)	2.5 mg/day for 4 weeks, 5 mg/day for 4 weeks, 10 mg/day for 18 weeks	Lung function, VEGF-D, 6MWD, everolimus pharmacokinetics, AEs.
Takada [37] (2016) Japan	Ann Am Thorac Soc	Multicenter, single-arm, open-label trial	LAM and TSC-LAM (*n* = 63)	2 years of treatment	Sirolimus (*n* = 63)	2 mg/day	Lung function, QOL scores, AEs
EI-Chemaly [38] (2017) USA	Chest	Two-center phase 1 trial	LAM (*n* = 13)	24 weeks of treatment, 24 weeks of observation	Hydroxychloroquine and sirolimus (*n* = 13)	Hydroxychloroquine (200 mg or 400 mg) Sirolimus (2 mg/day)	Lung function, 6MWD, QOL, AMLs, VEGF-D, AEs.
Author, Publication Year, Country	Journal	Study design	Diagnosis, No of patients	Study duration	Intervention and sample size	Treatment regimen	Study outcomes
Bee [9] (2018) UK	Thorax	A prospective national cohort, single-arm study,	LAM (*n* = 38), TSC-LAM (*n* = 9)	35.8 ± 18 months of treatment	Sirolimus (*n* = 47)	1–2 mg/day	Lung function, VEGF-D, AEs.
Cai [39] (2018) China	Orphanet J Rare Dis	Single-center, nonrandomized, open-label phase 2 trial	TSC-LAM (*n* = 6), TSC (*n* = 12)	1 year of treatment 1 year of observation	Everolimus (*n* = 18)	10 mg/day	AMLs, skin lesions, lung function, AEs.
Aghaeimeybodi [40] (2019) Iran	Caspian J Intern Med	A prospective phase 1 trial	LAM (*n* = 2), TSC-LAM (*n* = 4)	1 year of treatment	Sirolimus (*n* = 6)	2 mg/day	Lung function, 6MWD, AEs.

The general information of the included studies, including: name of the first author, publication year, study location, name of the journal, study design, number and diagnosis of patients, study duration, intervention and sample size of it, treatment regimen, and outcome indicator type for each study

*LAM* lymphangioleiomyomatosis, *TSC* tuberous sclerosis complex, *AML* angiomyolipomas, *6MWD* 6-min walk distance, *AEs* adverse events, *IM* Intramuscular injection*, VEGF-D* vascular endothelial growth factor D, *QOL* quality of life, *MMP* matrix metalloproteinase

### Effects on FEV_1_, FVC, DLco, 6MWD and QOL scores

**Fig. 2:**
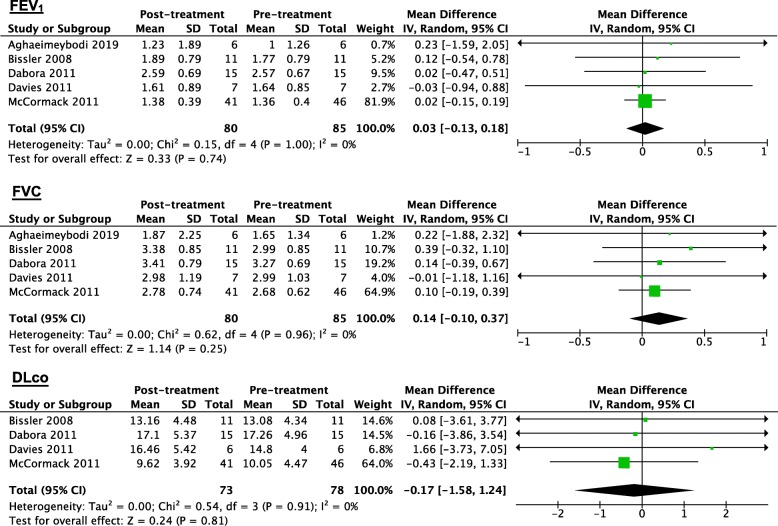
Forest plot for the weighted mean difference of FEV_1_, FVC and DLco with 95% confidence intervals in LAM patients treated with sirolimus. The weighted mean difference values of FEV_1_, FVC and DLco were 0.03 L (95% CI: − 0.13 to 0.18), 0.14 L (95% CI: − 0.10 to 0.37,) and − 0.17 ml/min/mmHg (95% CI: − 1.58 to 1.24), respectively. But the changes of them were not statistically significant, for all the *P* values of test for overall effect were above 0.05 (*P* = 0.74, *P* = 0.25 and *P* = 0.81), respectively

In LAM patients treated with sirolimus, the results of this meta-analysis showed that the changes in the FEV_1_, FVC, DLco and 6MWD values from baseline to the endpoint were not statistically significant. The WMD values of FEV_1_, FVC, DLco and 6MWD were 0.03 L (95% CI: − 0.13 to 0.18, *P* = 0.74, *I*^*2*^ = 0%, Fig. 2), 0.14 L (95% CI: − 0.10 to 0.37, *P* = 0.25, *I*^*2*^ = 0%, Fig. 2), − 0.17 ml/min/mmHg (95% CI: − 1.58 to 1.24, *P* = 0.81, *I*^*2*^ = 0%, Fig. 2) and 23.76 m (95% CI: − 12.96 to 60.47, *P* = 0.20, *I*^*2*^ = 0%, Figure A1), respectively. The endpoint values for lung function and the 6MWD were measured after 1 year [24, 33, 34, 40] or 2 years [35] of sirolimus treatment. In addition, two studies reported the changes in the Visual Analogue Scale (VAS) scores and total Functional Performance Inventory (FPI) scores for LAM patients treated with sirolimus. Compared with the placebo group, the sirolimus group had significant improvements in the VAS and FPI scores [33]. However, Takada et al. [37] did not detect significant changes in the VAS and FPI scores in LAM patients after 2 years of sirolimus treatment. In addition, the dosages of sirolimus treatment were variable: 0.25 mg/m^2^ body surface area [24], 0.5 mg/m^2^ body surface area [35] and 1–2 mg/day [33, 34, 37, 38, 40].

**Fig. 3:**
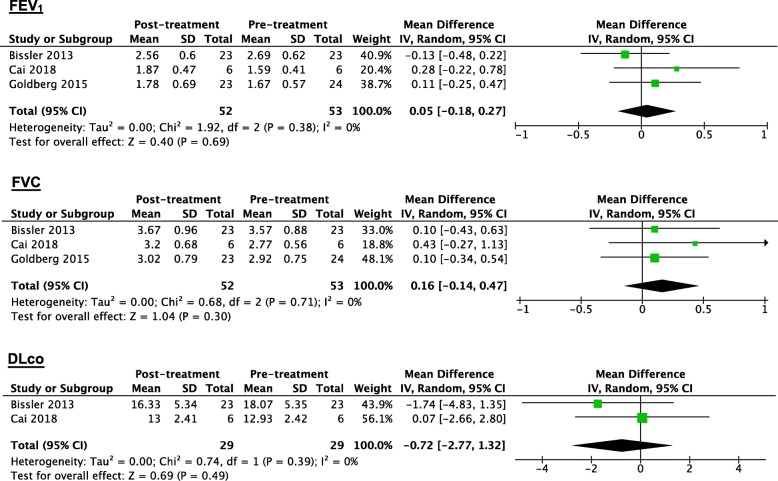
Forest plot for the weighted mean difference of FEV_1_, FVC and DLco with 95% confidence intervals in LAM patients treated with everolimus. The weighted mean difference values of FEV_1_, FVC and DLco were 0.05 L (95% CI: − 0.18 to 0.27), 0.16 L (95% CI: − 0.14 to 0.47,) and − 0.72 ml/min/mmHg (95% CI: − 2.77 to 1.32), respectively. But the changes of them were not statistically significant, for all the P values of test for overall effect were above 0.05 (*P* = 0.69, *P* = 0.30 and *P* = 0.49), respectively

In LAM patients treated with everolimus, the results also showed that the changes in the FEV_1_, FVC and DLco values from baseline to the endpoint were not statistically significant. The WMD values of FEV_1_, FVC and DLco were FEV_1_: 0.05 L (95% CI: − 0.18 to 0.27, *P* = 0.69, *I*^*2*^ = 0%), FVC: 0.16 L (95% CI: − 0.14 to 0.47, *P* = 0.30, *I*^*2*^ = 0%) and DLco: − 0.72 ml/min/mmHg (95% CI: − 2.77 to 1.32, *P* = 0.49, *I*^*2*^ = 0%), respectively (Fig. 3). Only Goldberg et al. [36] reported that the mean increase in the 6MWD was 47 m (95% CI: − 0.30 to 97.0) after 26 weeks of everolimus treatment, although the *P* value of this change was not provided. The everolimus treatment regimen in the included studies was 10 mg/day with a duration from 26 weeks to 1 year [25, 36, 39]. No prospective study has reported the effect of everolimus treatment on QOL scores.

**Fig. 4:**
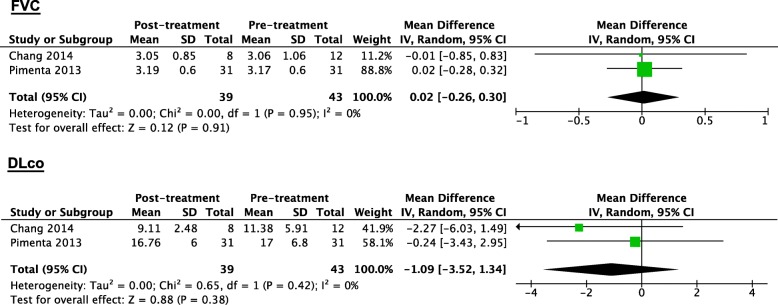
Forest plot for the weighted mean difference of FVC and DLco with 95% confidence intervals in LAM patients treated with doxycycline. The weighted mean difference values of FVC and DLco were 0.02 L (95% CI: − 0.26 to 0.30) and − 1.09 ml/min/mmHg (95% CI: − 3.52 to 1.34), respectively. But the changes of them were not statistically significant, for all the P values of test for overall effect were above 0.05 (*P* = 0.91and *P* = 0.38)

In LAM patients treated with doxycycline, neither FVC nor DLco values changed significantly according to the results of meta-analysis, with WMD values of 0.02 L (95% CI: − 0.26 to 0.30, *P* = 0.91, *I*^*2*^ = 0%) and − 1.09 ml/min/mmHg (95% CI: − 3.52 to 1.34, *P* = 0.38, *I*^*2*^ = 0%), respectively (Fig. 4). Additionally, Pimenta et al. [22] reported that after 12 months of doxycycline treatment (100 mg/day), there was a significant mean decrease in the FEV_1_ (− 70 ml, *P* = 0.034) and a significant median increase in the mental health scores (18, *P* = 0.006) on the Medical Outcomes Study 36-item Short-form Health Survey (SF-36). No significant variation in the 6MWD or other domains of the SF-36 was identified. In an RCT by Chang et al. [23], the authors reported that there was no significant difference in the changes in the St. George’s Respiratory Questionnaire (SGRQ) scores between the doxycycline treatment group (200 mg/day) and the placebo group after 24 months of treatment.

Apart from the treatment mentioned above, triptorelin, a gonadotrophin-releasing hormone analog, was reported to have no significant effect on preventing the decline in lung function in LAM patients in a prospective single-arm study [20]. In addition, the use of triptorelin might be associated with the loss of bone mineral density. EI-Chemaly et al. [38] reported that after 24 weeks of combination therapy (sirolimus 2 mg/day and hydroxychloroquine 200 mg–400 mg), the FEV_1_ and 6MWD values increased significantly (*P* < 0.05) but subsequently declined toward the baseline values and were significantly different at the end of treatment (*P* < 0.05). However, the FVC and DLco values and the SGRQ score were not significantly changed throughout the combination treatment period.

### Effects on AML and VEGF-D levels

**Fig. 5:**
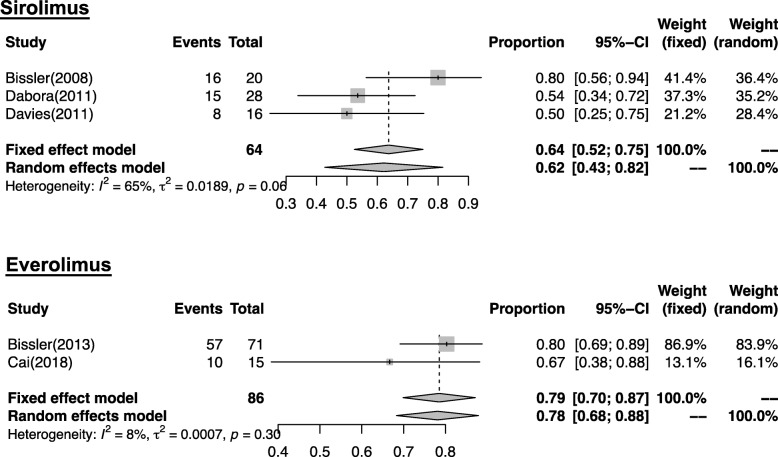
Forest plot for the pooled response rate of AML with 95% confidence intervals in LAM patients treated with sirolimus or everolimus. Random effects model is suitable for single-arm analysis. The pooled response rate of AML for LAM patients treated with sirolimus or everolimus was 0.62 (95% CI: 0.43 to 0.82) and 0.78 (95% CI: 0.68 to 0.88), respectively

The response rate of AML was available in 5 studies [24, 25, 34, 35, 39]. Among them, 4 studies provided the numbers of patients for whom the AML volume was reduced by at least 30% or more [24, 25, 34, 35], and 1 study provided the proportion of patients who achieved a ≥ 50% reduction in the AML volume [39]. In the random effect model, the pooled response rate of AML for LAM patients treated with sirolimus or everolimus was 0.62 (95% CI: 0.43 to 0.82, *I*^*2*^ = 65%) and 0.78 (95% CI: 0.68 to 0.88, *I*^*2*^ = 8%), respectively (Fig. 5).

Three articles provided the exact values of the VEGF-D levels in LAM patients before and after pharmacological treatment, including 2 studies describing doxycycline treatment [22, 23] and 1 study describing sirolimus treatment [33]. The meta-analysis showed no significant difference in VEGF-D levels before and after doxycycline treatment, with a WMD of − 2.65 pg/ml (95% CI: − 596.89 to 591.59, *P* = 0.99, *I*^*2*^ = 0%, Figure A2). However, McCormack et al. [33] found that the VEGF-D levels in 46 LAM patients decreased from 1848 ± 1514 pg/ml to 862 ± 540 pg/ml (*P* = 0.001) after 1 year of sirolimus treatment. Although no detailed VEGF-D values in LAM patients were provided, Dabora et al. [34] also reported a decrease in VEGF-D levels in TSC-LAM and TSC patients treated with sirolimus. The same trend was also reported in 2 articles describing everolimus treatment in LAM, TSC-LAM and TSC patients [25, 36]. Similar findings have also been documented in LAM patients treated with a combination of sirolimus and hydroxychloroquine [38].

### Safety events associated with pharmacological treatments for LAM patients

With regard to sirolimus treatment, the exact number of patients experiencing AEs was available in 4 studies [24, 34, 35, 40]. The common AEs during treatment were oral mucositis (60%), hyperlipidemia (43%), upper respiratory infection (29%), proteinuria (26%), urinary system infection (18%), headache (18%), peripheral edema (18%) and diarrhea (16%) (Figure A3).

Data regarding the number of patients experiencing AEs were provided in 3 studies describing everolimus treatment for LAM [25, 36, 39]. The most common AE was oral mucositis (75%) (Figure A4). Other common AEs included headache (40%), abdominal or flank pain (39%), hyperlipidemia (36%), diarrhea (31%), hypercholesterolemia (30%), acne (24%), upper respiratory infection (23%), nasopharyngitis (23%), cough (22%) and fatigue (20%) (Figure A4).

With regard to AEs in patients treated with doxycycline, only 2 studies provided detailed data [22, 23]. Headache (25%) and diarrhea (14%) were the two most common AEs reported in both of the studies (Figure A5). In addition, nausea (19%) and upper respiratory infection (83%) were also common AEs reported in the included studies.

Regarding LAM patients treated with triptorelin, flushing (36%), arthralgias and paresthesias (18%), and fatigue and dizziness (18%) were the common AEs reported by Harari et al. [20]. In LAM patients treated with a combination of sirolimus and hydroxychloroquine, oral mucositis (62%), headache (62%) and diarrhea (62%) were the most commonly reported AEs [38].

Notably, most of the AEs mentioned in the included studies were reported to be low or moderate grade and were tolerable.

### Sensitivity analysis

Sensitivity analysis confirmed that the exclusion of each study in turn did not change the meta-analysis results regarding lung function, the 6MWD, and VEGF-D levels. For sirolimus treatment, the data from Bissler et al. [24] were the main source of heterogeneity in the analyses of the AML response rate and AEs. Omitting that study [24], the *I*^*2*^ index of the AML response rate and the oral mucositis, proteinuria and diarrhea rates decreased to 0, 1.7, 0 and 0%, respectively. As for everolimus, the data by Cai et al. [39] were the main source of heterogeneity in the analyses of the acne and upper respiratory infection rates. After omitting these results, the *I*^*2*^ index decreased to 0 and 0%, respectively.

## Discussion

The impairment of lung function in LAM patients has been reported to be associated with the aberrant infiltration of LAM cells or MMPs released from LAM cells [42, 43]. The abnormal proliferation of LAM cells is due to the activation of the mTOR signaling pathway, which is induced by inactivating mutations in the TSC1/2 gene [13, 14, 44, 45]. Therefore, mTOR inhibitors may be effective in the treatment of LAM.

Consistent with the speculation mentioned above, the prospective trials included in our study have reported the efficacy of sirolimus in the improvement or stabilization of lung function, amelioration of QOL scores, and reduction in AML volume [9, 24, 33–35, 37, 38, 40]. Furthermore, 1 observational study [46] and 7 case reports [47–53] also addressed the efficacy of sirolimus in the management of chylous effusions. In the present study, quantitative analyses of lung function in patients treated with sirolimus revealed that the changes in lung function and 6MWD values were not significant. These results suggest that sirolimus is effective at stabilizing the lung function in LAM patients. However, there is not enough strong evidence to support the effect of this drug on improving lung function. Other reported benefits of sirolimus therapy were the reduction in AML volume and decrease in VEGF-D levels. The pooled response rate of AML (reduced by at least 30%) was 0.62 (95% CI: 0.43 to 0.82), but there were not enough raw data regarding the changes in VEGF-D levels for a meta-analysis. Therefore, this study could not assess the role of VEGF-D in measuring treatment efficacy.

Everolimus is another type of mTOR inhibitor that has also been used in the treatment of LAM. Compared with sirolimus, everolimus has a shorter half-life and better bioavailability [54]. In addition, exposure to everolimus can be terminated faster than that to sirolimus when a transplant procedure is planned [36]. Therefore, everolimus is an attractive potential option for LAM patients on the list for lung transplantation. The quantitative analyses in our study also indicated similar changes observed during sirolimus therapy in FEV_1_, FVC and DLco values during the application of everolimus. Among the included trials, only Cai et al. [39] reported the effects of everolimus with respect to improvements in both the FEV_1_ and FVC values, but the sample size was small: only 6 LAM patients. Goldberg et al. [36] also reported the improvement of FEV_1_ values during everolimus treatment, but the *P* value was not available. Therefore, it is still difficult to confirm the actual effects of everolimus on lung function in LAM patients. Nonetheless, 2 prospective studies [25, 39] reported the effect of everolimus in reducing in the volume of AMLs, and the pooled response rate was 0.78 (95% CI: 0.68 to 0.88).

Doxycycline is a tetracycline antibiotic that inhibits the production and activity of MMPs, which are overexpressed in serum and lung tissue in LAM patients near the cyst area and may contribute to cyst formation. However, doxycycline therapy was only demonstrated to be effective in improving lung function and QOL scores in a subgroup of 13 LAM patients with less severe disease. Notably, the effects of doxycycline were more likely to be due to the better baseline lung function of these patients [22, 55]. In contrast, the results from an RCT [23] and our quantitative analyses showed that doxycycline did not affect any outcome in LAM patients.

Only 1 prospective study reported triptorelin, a gonadotrophin-releasing hormone analog, in the treatment of LAM. The results showed no beneficial outcomes during triptorelin therapy and indicated that the application of triptorelin may be associated with some AEs [20]. Regarding the combination therapy, no strong evidence has been found to support its superiority over monotherapy in the treatment of LAM [38].

Generally, the prospect is still not optimistic in the treatment of LAM. Only sirolimus and everolimus have been reported to be effective for LAM in prospective studies. In addition, the results of the current meta-analysis support only the opinion that these drugs are effective at stabilizing the condition. Although sirolimus has been recommended for the treatment of LAM [1], several problems still exist in its application. Questions about when and how much sirolimus should be used to treat which kind of LAM still needs to be answered.

With respect to patient selection and the timing of treatment initiation, Bee et al. [9] found that LAM patients with better pretreatment lung function and shorter disease duration have a better response to sirolimus. However, the MILES study [56] suggested that severe LAM patients with higher VEGF-D levels may benefit more from sirolimus treatment. Furthermore, although sirolimus was suggested for application in LAM patients with an annual FEV_1_ loss of 90 ml/year (which is threefold higher than the normal FEV_1_ loss) in one guideline [1], whether it should be applied in asymptomatic patients with normal or mildly impaired lung function is still unclear [57, 58]. Regarding the treatment dosage, Bee et al. [9] also found that different serum levels of sirolimus produced similar effects, and lower doses had fewer side effects. However, these results should be interpreted with caution because the sample size in each dosage group was quite small. The dose-response curve for sirolimus remains unclear, and the optimal dose with the fewest AEs and sufficient effects remains uncertain. With respect to treatment duration, two prospective studies have reported that the effects of sirolimus were only present during its administration; the decline in lung function and growth of AMLs resumed when treatment was stopped [24, 33]. Although Takada et al. [37] and Yao et al. [59] have reported the durable safety and efficacy of sirolimus (the mean durations of sirolimus treatment were 2 years and 4.6 years, respectively), the optimal treatment duration and whether the long-term application of sirolimus could reduce the need for lung transplantation or even replace it are still unknown. Moreover, similar questions also existed during the use of everolimus in LAM patients. It is hoped that two ongoing multicenter international trials will help us better understand the above issues (ClinicalTrials.gov identifiers NCT03150914 and NCT02432560).

Our study had several limitations. First, because LAM is a rare disease, the number and sample sizes of the included studies were limited. Only 3 RCTs were available, and the other studies were single-arm designed without a placebo control group. These factors could have added bias to this meta-analysis. Second, five studies that included TSC patients cofounded the evaluations of AEs and the response rate of AML. Third, the data of the included studies were unavailable for subgroup analyses to assess the impacts of certain variables. Finally, only three pharmacological treatments were quantitatively analyzed due to the lack of raw data.

## Conclusions

In conclusion, our systematic review and meta-analysis support the application of sirolimus and everolimus in LAM, as these medications may stabilize lung function and alleviate renal AML. Further studies are required to reveal the optimal timing, duration and dosage in the application of sirolimus or everolimus. Doxycycline and triptorelin were not recommended for LAM because no beneficial outcomes were consistently reported. The efficacy and safety of combination therapy still require further exploration.

## Supplementary information

**Additional file 1.**

## Data Availability

All data generated or analyzed during this study are included in this published article (and its supplementary information files).

## Abbreviations

6MWD: 6-min walk distance
AEs: Adverse events
AML: Angiomyolipoma
CI: Confidence interval
DLco: Diffusion coefficient for carbon monoxide
FEV_1_: Forced expiratory volume in 1 s
FPI: Functional performance inventory
FVC: Forced vital capacity
LAM: Lymphangioleiomyomatosis
MMP: Matrix metalloproteinase
mTOR: Mammalian target of rapamycin
QOL: Quality of life
RCT: Randomized controlled trial
SGRQ: St George’s Respiratory Questionnaire
TSC: Tuberous sclerosis complex
VAS: Visual analogue scale
VEGF-D: Vascular endothelial growth factor D
WMD: Weighted mean difference
