# Supplementary material for: The efficacy and safety of pharmacological treatments for lymphangioleiomyomatosis
Source: Respir Res. 2020 Feb 14;21:55. doi: 10.1186/s12931-020-1316-3 (PMC7023761; doi:10.1186/s12931-020-1316-3)
Supplement: Supplementary file 1 — Additional file 1. [file 12931_2020_1316_MOESM1_ESM.docx]

**Additional file**

**The efficacy and safety of pharmacological treatments for lymphangioleiomyomatosis**

Qi Wang^1^, Mengqi Luo^2^, Bo Xiang^1^, Siyuan Chen^3^, Yi Ji^1^

^1^ Division of Oncology, Department of Pediatric Surgery, West China Hospital of Sichuan University, Chengdu, China.

^2^ State Key Laboratory of Oral Diseases, National Clinical Research Centre for Oral Diseases, West China Hospital of Stomatology, Sichuan University, Chengdu, China.

^3^ Pediatric Intensive Care Unit, Department of Critical Care Medicine, West China Hospital of Sichuan University, Chengdu, China.

Correspondence: Dr Ji Yi MD, PhD; Division of Oncology, Department of Pediatric Surgery, West China Hospital of Sichuan University, #37# Guo-Xue-Xiang, Chengdu 610041, China. Email: jijiyuanyuan@163.com

| Author, Publication year | Random sequence generation | Allocation concealment | Blinding of participants and personnel | Blinding of outcome assessment | Incomplete outcome data | Selective reporting | Other bias |
| --- | --- | --- | --- | --- | --- | --- | --- |
| McCormack,2011 | unclear risk of bias | low risk of bias | low risk of bias | low risk of bias | low risk of bias | low risk of bias | unclear risk of bias |
| Bissler,2013 | unclear risk of bias | low risk of bias | low risk of bias | low risk of bias | low risk of bias | high risk of bias | unclear risk of bias |
| Chang,2014 | unclear risk of bias | low risk of bias | low risk of bias | unclear risk of bias | low risk of bias | low risk of bias | unclear risk of bias |

**Table A1** Quality assessment for randomized controlled trials by Cochrane risk of bias assessment tool.

**Table A2** Quality assessment for single-arm trials by the methodological index for non-randomized studies (MINORS).

| Author,  publication year | A clearly stated aim | Inclusion of consecutive patients | Prospective collection of data | Endpoints appropriate to the aim of the study | Unbiased assessment of the study endpoint | Follow-up period appropriate to the aim of the study | Loss to follow up less than 5% | Prospective calculation of the study size |
| --- | --- | --- | --- | --- | --- | --- | --- | --- |
| Bissler,2008 | 2 | 2 | 2 | 2 | 0 | 2 | 0 | 2 |
| Harari,2008 | 2 | 0 | 2 | 2 | 0 | 2 | 0 | 2 |
| Dabora,2011 | 2 | 2 | 2 | 2 | 0 | 2 | 0 | 2 |
| Davies,2011 | 2 | 2 | 2 | 2 | 0 | 2 | 0 | 2 |
| Pimenta,2013 | 2 | 2 | 2 | 2 | 0 | 2 | 0 | 2 |
| Goldberg,2015 | 2 | 2 | 2 | 2 | 0 | 2 | 0 | 2 |
| Takada,2016 | 2 | 2 | 2 | 2 | 0 | 2 | 0 | 0 |
| EI-Chemaly,2017 | 2 | 2 | 2 | 2 | 0 | 2 | 0 | 2 |
| Bee, 2018 | 2 | 2 | 2 | 2 | 0 | 2 | 2 | 0 |
| Cai, 2018 | 2 | 2 | 2 | 2 | 0 | 2 | 0 | 2 |
| Aghaeimeybodi,2019 | 2 | 1 | 2 | 2 | 0 | 2 | 0 | 2 |

The items are scored 0 (not reported), 1 (reported but inadequate), 2 (reported and adequate).

**Figure.A1** Forest plot for the weighted mean difference of 6MWD with 95% confidence intervals in LAM patients treated with sirolimus.

The weighted mean difference value of 6MWD in LAM patients treated with sirolimus was 23.76 m (95% CI: -12.96 to 60.47). But the change was not statistically significant, for the P values of test for overall effect was above 0.05 (P = 0.20).

**Figure. A2** Forest plot for the weighted mean difference of VEGF-D level with 95% confidence intervals in LAM patients treated with doxycycline.

The weighted mean difference value of VEGF-D level in LAM patients treated with doxycycline was -2.65 pg/ml (95% CI: -596.89 to 591.59). But the change was not statistically significant, for the P values of test for overall effect was above 0.05 (P = 0.99).

**Figure. A3** Forest plot of the pooled proportions of AEs with 95% confidence intervals in LAM patients treated with sirolimus, ‘n’ represents the number of included study.

**Figure. A4** Forest plot of the pooled proportions of AEs with 95% confidence intervals in LAM patients treated with everolimus, ‘n’ represents the number of included study.

**Figure. A5** Forest plot of the pooled proportions of AEs with 95% confidence intervals in LAM patients treated with doxycycline, ‘n’ represents the number of included study.
